# Periodic fever, aphthous stomatitis, pharyngitis and cervical adenitis syndrome in children: a brief literature review

**DOI:** 10.1590/1984-0462/2022/40/2021087IN

**Published:** 2022-06-10

**Authors:** Cristina Terumy Okamoto, Hanne Lise Chaves, Mateus José Schmitz

**Affiliations:** a Universidade Positivo, Curitiba, PR, Brazil.

**Keywords:** Relapsing fever, Stomatitis, aphthous, Pharyngitis, Lymphadenitis, Child, Hereditary autoinflammatory diseases, Febre recorrente, Estomatite aftosa, Faringite, Adenite, Crianças, Doenças hereditárias autoinflamatórias

## Abstract

**Objective:**

To describe clinical, diagnostic and therapeutic characteristics of the periodic fever, aphthous stomatitis, pharyngitis and cervical adenitis (PFAPA) syndrome.

**Data source:**

Literature review in the PubMed database by using specific descriptors to identify all articles published in the English language in the last three years; 38 articles were found. After performing selection of titles and abstract analysis, 13 out of the 38 articles were fully read. Relevant studies found in the references of the reviewed articles were also included.

**Data synthesis:**

The PFAPA syndrome (Periodic Fever, Aphthous Stomatitis, Pharyngitis and cervical Adenitis) is a medical condition grouped among the periodic fever syndromes. The etiology is uncertain, but possibly multifactorial, and its symptoms are accompanied by recurrent febrile episodes although weight and height development are preserved. It is a self-limiting disease of benign course with remission of two to three years without significant interference in the patient's overall development. Treatment consists of three pillars: interruption of febrile episodes, increase in the interval between episodes, and remission.

**Conclusions:**

Despite several attempts to establish more sensitive and specific criteria, the diagnosis of PFAPA syndrome is still clinical and reached by exclusion, based on the modified Marshall's criteria. The most common pharmacological options for treatment include prednisolone and betamethasone; colchicine may be used as prophylaxis, and surgical treatment with tonsillectomy can be considered in selected cases.

## RESUMO

**Objetivo:**

Descrever as características clínicas, diagnósticas e de tratamento da síndrome de febre periódica, estomatite aftosa, faringite e adenite (PFAPA).

**Fontes de dados:**

Revisão de literatura na base de dados PubMed, feita por meio de descritores específicos para identificar todos os artigos publicados em língua inglesa nos últimos três anos. Dos 38 artigos encontrados, foram encaminhados para leitura integral 13 publicações após seleção de títulos e análise de *abstract*. Estudos relevantes encontrados nas referências dos artigos revisados também foram incluídos.

**Síntese dos dados:**

A PFAPA é traduzida do inglês *periodic fever*, *aphthous stomatitis*, *pharyngitis and cervical adenitis*. Caracterizada por etiologia ainda incerta e possivelmente multifatorial, seus sintomas são acompanhados por episódios recorrentes de febre associados a um desenvolvimento pôndero-estatural preservado. É uma doença autolimitada de curso benigno, com remissão em dois a três anos, sem interferências significativas no desenvolvimento do paciente pediátrico. O tratamento consiste em três pilares: interrupção da crise febril, aumento do intervalo entre crises e remissão.

**Conclusões:**

Apesar de várias tentativas de estabelecer critérios atuais mais sensíveis e específicos, o diagnóstico da síndrome PFAPA ainda é clínico e de exclusão com base nos critérios de Marshall modificados. As opções farmacológicas mais utilizadas para o tratamento são a prednisolona e betametasona; colchicina pode ser utilizada como profilaxia e o tratamento cirúrgico com tonsilectomia pode ser considerado em casos selecionados.

## INTRODUCTION

Periodic fever, aphthous stomatitis, pharyngitis and cervical adenitis, whose acronym is PFAPA, is an autoinflammatory syndrome described by Marshall et al.^1^ It is a diagnosis of exclusion, based on clinical criteria, and it is worth noting that the weight and height development of patients is not affected.^2^ estomatite aftosa, faringite e adenite (PFAPA

In children, PFAPA syndrome is the most common of the periodic fever syndromes.^3^ Its exact etiopathogenesis is still unknown, but a genetic basis is suggested, especially when considering the familial occurrence.^4,5^

The objective of this literature review is to describe the most important concepts in relation to the etiopathogenesis, clinical pattern, diagnosis, and treatment of PFAPA syndrome in pediatric practice.

## DATA SOURCE

A literature review was carried out with a search in the electronic database U.S. National Library of Medicine (PubMed), using the descriptors “periodic fever AND PFAPA AND children” to identify all articles published in the English language in the last three years. A total of 38 articles were found. After the selection of titles, 14 publications that did not fit the research scope were excluded, and the abstracts of 24 publications were read. A total of 11 studies were excluded, whose objectives did not meet those of the present research, and 13 publications were fully read. Relevant studies found in the references of the reviewed articles were also included.

## DATA SYNTHESIS

### Etiopathogenesis: an overview

The PFAPA syndrome has been extensively studied, but the exact mechanisms of its pathophysiology remain unclear.^4–7^ It has been shown that several pro-inflammatory cytokines participate in the development of PFAPA episodes, such as interferon, tumor necrosis factor, and interleukins,^7,8^ and there is also evidence that cellular immunity may play a role in the syndrome.^9^

Furthermore, autoinflammatory diseases have a genetic basis.^7^ The review conducted by Wekell cites several studies that sought to demonstrate such bases and that would explain the syndrome, but the results are controversial and it has not yet been possible to prove what exactly is the type of inheritance or the gene linked to PFAPA. It is suggested that this inheritance is polygenic or multifactorial, at least in most cases.^5,7^

A North American case-control study described the prevalence of family history and inherited patterns of patients with PFAPA syndrome, and showed that parents of children diagnosed with PFAPA were more likely to have recurrent pharyngitis or PFAPA (36 *vs.* 16%; p<0.001), aphthous stomatitis (46 *vs.* 28%; p=0.002), or a combination of recurrent pharyngitis and aphthous stomatitis (22 *vs.* 7%; p<0.001) compared with parents of healthy children.^10^

In addition to the aforementioned aspects, it is known that tonsils play a well-defined role in the pathophysiology of the syndrome due to the remission of symptoms after tonsillectomy.^5,11^ A recent, multicenter study analyzed a series of 23 cases of children diagnosed with PFAPA and who underwent tonsillectomy, with follow-up for 12 months after surgery. Complete remission of symptoms was observed in 21 children soon after the procedure, and in two children after three months.^12^ Although it is well-established that tonsillectomy is an effective treatment option, the exact pathophysiology of its role in the disease remains unclear.^5,12^

Recently, the role of vitamin D in the course of PFAPA syndrome has been assessed.^13^ Studies have already shown that it plays an important role in regulating the immune system and, therefore, it may play a role in autoinflammatory syndromes.^14,15^

A recent, prospective study evaluated the serum vitamin D level (25[OH]D) of children diagnosed with PFAPA and compared it with that of healthy children in the control group; a significantly lower concentration was observed in children with PFAPA (18±10 *vs.* 35±13ng/mL; p<0.001). Multivariate logistic regression analyses showed that serum 25(OH)D levels lower than 30ng/mL were associated with the occurrence of PFAPA syndrome (*Odds Ratio* [OR] 2.1; 95% confidence interval [95%CI] 1.8–2.5). Nevertheless, the study concludes that hypovitaminosis D may be a risk factor for the recurrence of symptoms.^16^

These results confirmed a previous analysis that had been performed with 25 Italian children who met the criteria for PFAPA syndrome. According to this research, patients with PFAPA had, in general, a lower serum level of 25(OH)D compared with the control group (16.5±7.3 *vs.* 29.8±8.4ng/mL; p<0.000). After supplementation with vitamin D (400 IU/day for seven months), a change in the pattern of febrile episodes was observed: the mean duration of each episode ranged from 4.3 to 2.3 days (95%CI 1.9–3 .1; p<0.05) and 36% of patients had a reduction in the number of episodes per year (reduction from 8.9±2.0 to 2.9±2.1 days; p<0.005).^17^

In addition to hypovitaminosis D, other hypotheses have been tested to discover possible risk factors for the syndrome. A recent case-control study found that children with PFAPA had lower breastfeeding rates compared with children in the control group (94 *vs.* 99%; OR 0.1 [95%CI 0.02–0.5]; p=0.006), and that more mothers of PFAPA patients were smokers compared with mothers in the control group (23 *vs.* 14%; OR 2.5 [95%CI 1.3–4.6]; p=0.005). These data remained statistically significant after controlling for the analyses of the socioeconomic status of the family. In other words, smoking and lack of breastfeeding were more common in patients with the syndrome than in healthy children in the control group.^6^ This association only reinforces the role of the environment in the development of PFAPA, evidencing its multifactorial nature.

### Diagnosis: an uncertainty

PFAPA syndrome usually presents with fever and nonspecific but homogeneous symptoms, which are aphthous stomatitis, pharyngitis, and cervical adenitis. Episodes usually last two to eight days, recur at intervals of two to 12 weeks, and alternate with asymptomatic intervals.^3,18^

For diagnosis, the modified Marshall's criteria (Chart 1) proposed by Thomas et al. are considered^19^. Despite being used for over 20 years, such criteria have not been validated by large clinical trials and are limited; they have good sensitivity, but lack specificity.

**Chart 1:** Modified Marshall's criteria for the diagnosis of periodic fever, aphthous stomatitis, pharyngitis, and cervical adenitis syndrome.

I. Onset of regular recurring fevers before five years of age
II. Symptoms in the absence of upper respiratory tract infection with at least one of the following signs:
– aphthous stomatitis
– cervical lymphadenitis
– pharyngitis
III. Exclusion of cyclic neutropenia
IV. Asymptomatic interval between fever episodes
V. Normal child development and growth

Adapted from Thomas.^19^

PFAPA is not a well-defined entity, it overlaps with other pathologies that also manifest with periodic fever and have an infectious or genetic origin, such as Familial Mediterranean Fever, whose treatment and prognosis greatly differ. International efforts have been made in recent years seeking to propose new diagnostic criteria that provide sufficient specificity for the diagnosis of the syndrome.

Notably, a study published by Vanoni et al. held a consultation with specialists in autoinflammatory diseases aiming to propose new diagnostic criteria (Chart 2). However, these criteria did not undergo multivariate statistical analyses, adjusted for possible confounding factors; they translate into a set of characteristics that can be used in future research to define criteria to be applied in daily clinical practice. The authors emphasize that these new criteria seem to be difficult to apply, as they require a precise and detailed anamnesis, which is not always possible to obtain from parents. In addition, the characterization of fever is probably too restrictive and excluded almost half of the patients, who, according to Marshall's criteria, would have been included; therefore, a significant number of patients may not be diagnosed. Therefore, they do not consist in useful diagnostic criteria in clinical practice.^20^

**Chart 2:** Classification criteria for the diagnosis of periodic fever, aphthous stomatitis, pharyngitis, and cervical adenitis syndrome according to Vanoni et al.^20^

I. Periodic fever for at least six months:
a. daily fever of at least 38.5°C (axillary temperature) for two to seven days
b. at least five episodes of recurrent fever no more than two months apart
II. At least one of the following symptoms in each episode and at least two of the three in most episodes:
– aphthous stomatitis
– cervical lymphadenitis
– pharyngitis
III. Clinical or laboratory exclusion of other causes of recurrent fever
IV. Exclusion of infections, immunodeficiency, and cyclic neutropenia
V. Onset of disease before six years of age VI. Full recovery between episodes

Adapted from Vanoni et al.^20^

Renko et al. carried out a literature review to verify if there is sufficient evidence to propose new diagnostic criteria for PFAPA. The authors observed that most children with the syndrome had their first fever episode between 11 months and two years of age; however, up to 20% of them have the first symptom after the age of five years. Regarding fever, it was observed that each febrile episode lasts for about seven days, during which the temperature is between 39 and 40°C. There is an increase in serum inflammatory markers, notably C-reactive protein, which is between 120 and 179mg/L during days when patients are affected by fever. Patients are asymptomatic between episodes, despite the loss of quality of life due to recurrent episodes.

The most common associated symptom is pharyngitis, which occurs in about 90% of patients, and cervical adenitis, in up to 93% of children. Aphthous stomatitis is less common, reaching about 50% of them. Other symptoms that may be present are nausea, diarrhea, and arthralgia. Therefore, the diagnostic criteria suggested by this study are: 1) history of at least five recurrent episodes of fever; 2) absence of another condition that could explain the fever; 3) risk assessment for cyclic neutropenia; 4) absence of symptoms between episodes; 5) normal weight and height development for the age.

These criteria are not yet validated for use in daily clinical practice, but they may assist in characterizing the syndrome.^21^

Takeuchi et al. carried out a prospective study whose objective was to establish new, more specific diagnostic criteria for PFAPA. The authors selected 257 children who met the modified Marshall's criteria and analyzed their clinical and laboratory characteristics, family history, and therapeutic response. A frequency of febrile episodes of at least four times a year was observed to have a sensitivity and specificity of, respectively, 98.0 and 80.8% in distinguishing PFAPA from an infectious episode; when the cutoff value was three episodes per year, it was difficult to distinguish the two situations.

The established criteria were based on the obtained results; the presence of four criteria provides 93.8% sensitivity and 94.2% specificity for infectious diseases; and 93.8 and 95.6%, respectively, for Familial Mediterranean Fever. When five items are present, the sensitivity in both cases is 100% and the specificity, 80.2% (Chart 3). If only four items are present, it is classified as probable PFAPA. The authors concluded that a cohort is necessary to assess the accuracy and usefulness of the criteria. There are still expectations that the identification of a gene responsible for the syndrome will add more specificity to its diagnosis.^7^

**Chart 3:** Diagnostic criteria for periodic fever, aphthous stomatitis, pharyngitis, and cervical adenitis syndrome according to Takeuchi et al.^7^

I. Essential criterion:
– temperature above 38°C that lasts less than eight days and with recurrence of at least four times in one year
II. Supporting criteria:
– onset before five years of age
– tonsillitis or pharyngitis with whitish plaques
– at least one of the following aspects concomitantly occurs with the other symptoms: aphthous stomatitis; cervical lymphadenitis; sore throat; vomiting; severe headache
– absence of cough
– family history of recurrent fever, tonsillitis, or tonsillectomy
– laboratory tests indicating inflammation (elevated C-reactive protein) during a febrile episode
– elevated serum levels of immunoglobulin D
– good response to the use of glucocorticoids

Adapted from Takeuchi et al.^7^

The main differential diagnosis, and the most difficult, is made with infectious diseases, mainly in the upper respiratory tract. Another group of diseases that present very similarly to PFAPA are monogenic autoinflammatory diseases and immunodeficiencies.^11,22^

### Management: from treatment of episodes to prophylaxis

Management must be individualized and mainly take into account the intensity of the damage to the child's quality of life. Frequent episodes tend to cause absenteeism from school and worsen the quality of life of patients and their parents.^23^ aphthous stomatitis, pharyngitis and cervical adenitis (PFAPA Thus, the goals of treatment are: to reduce symptoms in the episode and to interrupt it; to prescribe a treatment for reducing the frequency of new episodes; and to induce remission.^22^

As a symptomatic treatment, non-steroidal anti-inflammatory drugs (NSAIDs) have better efficacy than the use of acetaminophen in the treatment of fever, although the use of ibuprofen and indomethacin has less significant efficacy for children with PFAPA.^24^ The main abortive treatment is the early administration of a single dose of corticosteroid during the febrile episode; prednisone is chosen at a dose of 1–2mg/kg/dose up to a maximum of 60mg or betamethasone at a dose of 0.1–0.2mg/kg/dose.^24–28^

Within a few hours, fever tends to disappear in more than 90% of patients, although some level of fatigue may continue for a few more hours or days.^29^ In the study conducted by Manthiram et al., it was found that many experts prescribe corticosteroids during a febrile episode, and 95% of them described it as an effective or very effective measure in controlling PFAPA symptoms.^30^

A randomized clinical trial of 41 patients with PFAPA tested a lower dose of prednisone at 0.5mg/kg/dose and noted that it took longer for reducing the fever (eight to 12 hours) compared with a dose of 2mg/kg/dose, which reduced the fever in six to eight hours. A second dose can be administered at home if there is rapid recurrence or low efficacy; a dose of 1mg/kg/dose of prednisone can be administered and reduced to the minimum effective dose.^31^ According to Federici et al., the use of corticosteroids usually interrupts the febrile episode in PFAPA and, in case this does not occur within a few hours, other differential diagnoses should be considered.^32^ It is noteworthy that other symptoms related to the oral cavity tend to disappear more slowly than fever.^33^

### Reduction in the frequency of episodes

Colchicine, which is the first treatment option for Familial Mediterranean Fever, seems to be an option to increase the interval between febrile episodes in PFAPA. It was effective in eight out of nine patients with the syndrome^34^ aphthous stomatitis, pharyngitis, adenitis, a datum confirmed in a randomized clinical trial with 18 patients, in which nine of them were treated with colchicine at an age-adjusted dose: children under five years of age received 0.5mg/day; those aged between five and ten years received 1mg/day; and those aged over ten years received 1.5mg/day. Of these, eight had significantly fewer febrile episodes than the control group (p≤0.01).^35^ randomized, controlled study among patients with PFAPA who attend the Pediatric Rheumatology Clinic at the Rambam Medical Center in Israel. A total of 18 patients aged 4-11 years (males:females ratio = 11:7

Colchicine increased the interval between episodes in 85% of patients in a Turkish study, in which 400 patients receiving the medication as prophylaxis were followed up for 12 months. There was an increase in the median from 20 to 50 days of interval, with a more pronounced effect on patients with heterozygous mutation in the gene for Mediterranean fever.^36^ aphthous stomatitis, pharyngitis and cervical adenitis (PFAPA Further studies are necessary to verify the efficacy of colchicine in preventing PFAPA in patients without mutation for Familial Mediterranean Fever.^22^

Individuals with PFAPA and low serum vitamin D levels may have higher levels of C-reactive protein and more frequent febrile episodes.^13^ Supplementation with 400IU/day of vitamin D reduced the duration and frequency of febrile episodes in these patients.^37^ Nevertheless, the prophylactically use of vitamin D still needs to be further studied.^22^

The use of probiotics, such as *Streptococcus salivarius* K12, may be beneficial, as it has been shown to reduce the frequency of pharyngotonsillitis and acute otitis media. In the case of patients with PFAPA, in one study, three out of four patients who used this probiotic for three months reported disappearance of symptoms and improved quality of life. However, there is need for a clinical trial to better assess these results.^24,27^ aphthous stomatitis, pharyngitis, and cervical adenopathy (PFAPA

### Surgical management and symptom remission

As an option to reduce the frequency of febrile episodes and even induce remission of the disease, tonsillectomy can be chosen. This suggestion was initially offered by Abramson et al., after noticing improvement after tonsillectomy in four patients.^38^ Subsequently, two randomized trials reported symptom remission in all patients who underwent the procedure (one study on 14 patients and another on 19 patients in the groups that underwent surgery).^39,40^

Burton et al. analyzed these two studies and could not conclude whether tonsillectomy could be useful in the treatment of PFAPA due to inclusion errors in the publications.^41,42^

A retrospective study concluded that the remission rate was significantly higher in patients who underwent tonsillectomy compared with those who were treated with methylprednisolone during the episode.^43^ But, in contrast, a 2010 meta-analysis found no significant difference in the effectiveness of corticosteroids and tonsillectomy.^44^

Another Turkish retrospective survey of 359 patients with PFAPA showed that complete clinical remission was achieved in 127 of 158 patients after the surgical procedure, and the persistence of symptoms in the group that underwent tonsillectomy was lower than in the group that did not undergo the procedure (p<0.05). The aforementioned study reported that the effectiveness of tonsillectomy was higher in patients who did not carry the Familial Mediterranean Fever gene.^45^ However, Lantto et al. evaluated 108 children who underwent tonsillectomy and had recurrent fever. The response to surgery in the group that did not meet the modified Marshall's criteria was similar to that of the group that met these criteria, in such a way that it can be concluded that tonsillectomy can be a good option even for patients who only present fever as a recurrent symptom.^46^ Tonsillectomy seems to be effective for the treatment of PFAPA; nonetheless, the literature is controversial as to its superiority over drug therapy.^22^

### Choosing the best approach

When choosing the aforementioned therapies, the risk and benefit of each one must be weighed, in addition to taking into account that, usually, the natural history of the disease is remission after two to three years, without long-term consequences. The main aspect that must be analyzed is the harmonious development of the child and their family relationship as well as their school performance.^22^

One to two doses of corticosteroids are usually effective for most children, and ten to 15 doses a year are usually safe. Colchicine is generally well-tolerated and may be a good option for children with febrile episodes every two to three weeks. Its use is aimed at prophylaxis, seeking to reduce the use of corticosteroids, but it can generate resistance in parents because it is a daily medication, especially when the abortive treatment during the episode is effective. Wekell et al. indicate using colchicine in children with atypical PFAPA syndrome, children who have not improved after tonsillectomy, and children whose aphthous stomatitis predominates.^47^

Surgical treatment with tonsillectomy should be considered for patients who do not respond to drug therapy and should take into account factors such as patient's age (not usually performed in patients under three years of age, in addition to not being very well-tolerated in adolescents) and concomitant conditions such as tonsillar hypertrophy or sleep apnea.^22^

In most children, the resolution of the disease spontaneously occurs within a few years, usually before puberty, although 20% of cases with initial improvement may have recurrence in adulthood.^24^ The prognosis of the disease is good and there are no long-term sequelae or association with comorbidities.^31,48^

PFAPA syndrome is an autoinflammatory syndrome with an unclear etiology. Its diagnosis is clinical and of exclusion; currently, the modified Marshall's criteria are still used. For its treatment, corticosteroids are usually administered, especially prednisone or betamethasone, as abortive treatments for the febrile episodes, which are effective in most patients.

To reduce the frequency of febrile episodes, colchicine is a good option. Surgical treatments, such as tonsillectomy, can also be evaluated, although evidence for their use in inducing remission is controversial.

The risk-benefit ratio of any treatment must always be considered, as the PFAPA syndrome has a benign evolution with good prognosis and usually has spontaneous remission within two to three years in most children, without harming their development or being at risk of future comorbidities.
